# Imaging of concomitant pulmonary and hepatic hydatid cysts in a young Ethiopian farmer

**DOI:** 10.1093/omcr/omaf202

**Published:** 2025-10-29

**Authors:** Elias Belete, Francesco Capriotti, Carolina Vitale, Carlo Airola

**Affiliations:** Medical Ward, St. Luke Catholic Hospital, Waliso, 00000, South-West Shewa Zone, Oromia, Ethiopia; Medical Ward, St. Luke Catholic Hospital, Waliso, 00000, South-West Shewa Zone, Oromia, Ethiopia; Medical Ward, St. Luke Catholic Hospital, Waliso, 00000, South-West Shewa Zone, Oromia, Ethiopia; Medical Ward, St. Luke Catholic Hospital, Waliso, 00000, South-West Shewa Zone, Oromia, Ethiopia

**Keywords:** Hydatid cyst, Ultrasonography, Liver echinococcosis, Pulmonary echinococcosis, Ethiopia

## Abstract

A 30-year-old female farmer from South-West Shewa, Ethiopia, presented with chest and abdominal pain. Imaging revealed concomitant pulmonary and hepatic cystic echinococcosis, with calcified liver cysts showing the classic cerebral gyri sign. According to WHO guidelines, pulmonary surgery was advised, while hepatic cysts warranted observation. Serology was unavailable, and the patient was lost to follow-up. This case highlights imaging as a key diagnostic tool and the need for preventive education in endemic settings.

A 30-year-old female farmer from South-West Shewa, Oromia, Ethiopia, presented with 3–4 weeks of dull right lower chest and upper abdominal pain. She reported no fever, cough, or systemic symptoms. The patient owns cattle and dogs, regularly handling livestock and dogs, including home slaughtering and feeding raw offal to dogs, common practices in her rural community [1, 2]. On medical evaluation, vital signs were normal and physical examination was unremarkable. Laboratory tests showed mild neutrophilic leukocytosis (WBC 13600/mm^3^; neutrophils 10 700/mm^3^); liver enzymes were normal. Echinococcus serology was unavailable, representing a limitation for the diagnosis.

Chest radiograph posteroanterior view (Fig. 1a) showed a well-circumscribed radio-opacity (approximately 2 cm) in the right lower lobe. A ring-calcified lesion (approximately 8 cm) was noted in the right upper abdominal quadrant. Abdominal ultrasound using a 3.7–4.5 MHz convex probe (Fig. 1b) identified two cystic lesions (6 cm and 8.5 cm) in hepatic segments VI and VII, respectively, with heterogeneous internal contents, partial wall calcification, and the classic ‘cerebral *gyri*’ sign.

**Figure 1:**
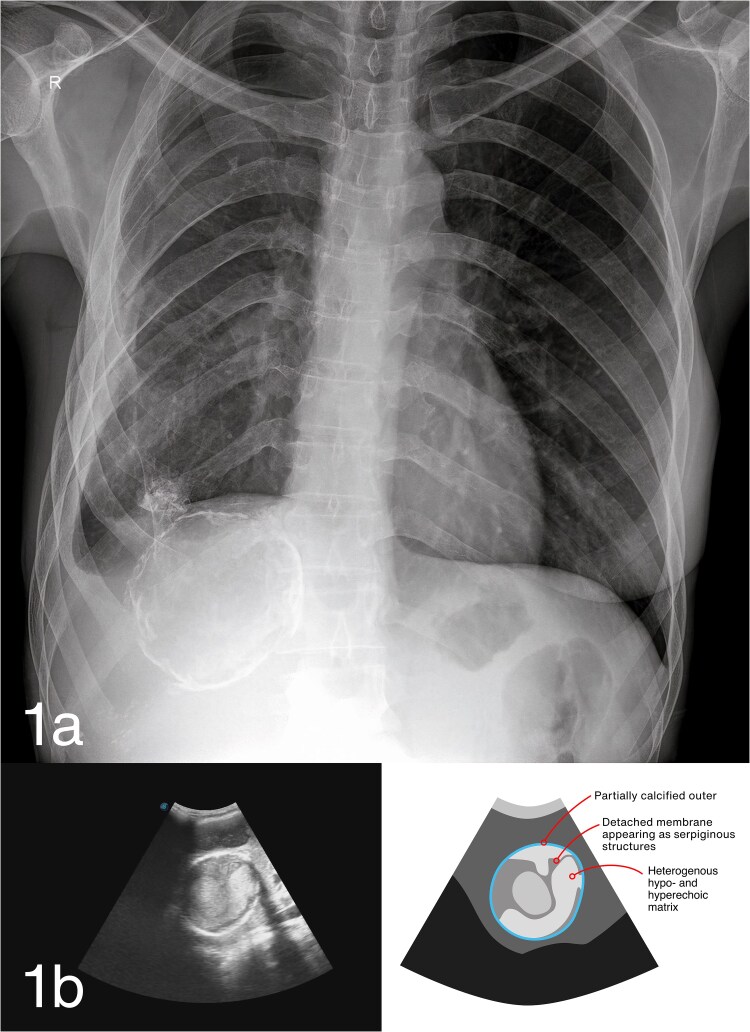
a) Chest X-ray showing a well-circumscribed opacity in the right lower lung and a ring-calcified lesion in the right upper abdominal quadrant; b) abdominal ultrasound showing hepatic hydatid cyst CE4 with internal non-homogeneous content, serpiginous structures (cerebral *gyri* sign), and partial calcification.

Hepatic hydatid cysts (WHO CE4) with probable pulmonary involvement were diagnosed. Differential diagnoses included hepatic tumors or abscesses, but the characteristic ultrasound appearance was diagnostic. She was referred for thoracic surgery because, according to WHO guidelines, pulmonary cysts require surgery before albendazole administration [3]. Follow-up is recommended for CE4 hepatic cysts (‘watch and wait’), as they are unlikely to contain viable protoscoleces but carry a risk of reactivation. The patient was lost to follow-up due to financial constraints. Hydatid disease caused by *Echinococcus granulosus* is endemic in Ethiopia. While local prevalence data are lacking for South-West Shewa, cystic echinococcosis has been reported at around 30% in bovine, 7.4% and 16,6% in caprine and ovine respectively, and 37% in dogs in other districts of Oromia [4]. Humans are accidental intermediate hosts that ingest eggs shed from dog feces. The liver is the most frequently affected organ, followed by the lungs. Simultaneous hepatic and pulmonary involvement occurs in 4–25% of cases [5]. Imaging findings, including calcified cyst walls and cerebral *gyri* sign, can guide diagnosis in resource-limited settings. This case highlights the importance of community education on safe livestock practices, early detection, and structured follow-up. Limitations include absence of serological confirmation and loss to follow-up.
